# Loss of TRPV4 Function Suppresses Inflammatory Fibrosis Induced by Alkali-Burning Mouse Corneas

**DOI:** 10.1371/journal.pone.0167200

**Published:** 2016-12-28

**Authors:** Yuka Okada, Kumi Shirai, Masayasu Miyajima, Peter S. Reinach, Osamu Yamanaka, Takayoshi Sumioka, Masahide Kokado, Katsuo Tomoyose, Shizuya Saika

**Affiliations:** 1 Ophthalmology, Wakayama Medical University, Wakayama, Japan; 2 Laboratory Animal Center, Wakayama Medical University, Wakayama, Japan; 3 Wenzhou Medical University School of Ophthalmology and Optometry, Wenzhou, P. R. China; Oklahoma State University Center for Health Sciences, UNITED STATES

## Abstract

In humans suffering from pulmonary disease and a mouse model, transient receptor potential vanilloid 4 (TRPV4) channel activation contributes to fibrosis. As a corneal alkali burn induces the same response, we determined if such an effect is also attributable to TRPV4 activation in mice. Accordingly, we determined if the alkali burn wound healing responses in wild-type (WT) mice are different than those in their TRPV4-null (KO) counterpart. Stromal opacification due to fibrosis in KO (n = 128) mice was markedly reduced after 20 days relative to that in WT (n = 157) mice. Immunohistochemistry revealed that increases in polymorphonuclear leukocytes and macrophage infiltration declined in KO mice. Semi-quantitative real time RT-PCR of ocular KO fibroblast cultures identified increases in proinflammatory and monocyte chemoattractant protein-1 chemoattractant gene expression after injury. Biomarker gene expression of fibrosis, collagen1a1 and α-smooth muscle actin were attenuated along with macrophage release of interleukin-6 whereas transforming growth factor β, release was unchanged. Tail vein reciprocal bone marrow transplantation between WT and KO chimera mouse models mice showed that reduced scarring and inflammation in KO mice are due to loss of TRPV4 expression on both corneal resident immune cells, fibroblasts and infiltrating polymorphonuclear leukocytes and macrophages. Intraperitoneal TRPV4 receptor antagonist injection of HC-067047 (10 mg/kg, daily) into WT mice reproduced the KO-phenotype. Taken together, alkali-induced TRPV4 activation contributes to inducing fibrosis and inflammation since corneal transparency recovery was markedly improved in KO mice.

## Introduction

Corneal transparency and an optically smooth curvature are both required for normal vision. On the other hand, an alkali injury induces opacification resulting in loss of transparency due to fibrosis, inflammation and neovascularization. There are therapeutic options available for reducing losses in corneal transparency in a clinical setting, but they can not inhibit fibrosis and they can have side effects as well as induce toxicity. Treatments include antibiotics, tear substitutes, corticosteroids, ascorbic acid, collagenase inhibitors, Mitomycin C, histone deacetylase inhibitors and surgical treatments such as penetrating keratoplasty and amniotic membrane transplantation [1]. However, the aforementioned limitations continue to prompt the search for novel treatment strategies to inhibit inflammatory fibrosis.

Transient receptor potential (TRP) channels constitute a superfamily of 28 genes that are subdivided into 7 subfamilies [2]. Each of these nonselective cation channels possess variable Ca^2+^ permeability and act as sensors by undergoing modulation in response to a wide array of inputs, including temperature, pressure, pH, voltage, chemicals, lipids, and other proteins [3, 4]. In the cornea, a number of these TRP channel subtypes in different subfamilies are involved in mediating responses that affect maintenace of corneal transparency. Two of the channels for which we identified functional roles are TRP vanilloid type 1 (TRPV1) and TRP ankyrin type 1 (TRPA1). Their activation by an alkali burn induced inflammatory fibrosis and neovascularization in a mouse corneal wound healing model. TRPV1 involvement in this undesirable wound healing outcome was verified by showing that in TRPV1 KO mice all of these sight compromising effects including stromal macrophage and/or polymorphonuclear leukocyte (PMNs) infiltration were reduced [5]. Another indicator of TRPV1 involvement is that in its absence eye globe contracture declined owing to blockage of transforming growth factor beta (TGFβ1)-induced myofibroblast transdifferentiation. This response induces opacification through TGFβ receptor transactivation of TRPV1 causing Ca^2+^ transients leading to p38 MAPK stimulation [6]. Regarding TRPA1, loss of its gene function also attenuates severe inflammation and fibrosis developing during wound healing. As in the TRPV1 study, intraperitoneal injection of different TRPA1 antagonists markedly suppressed excessive chronic inflammation and resultant tissue fibrosis induced by corneal alkali burning in mice [7, 8]. These results suggest that TRPV1 and TRPV4 are potential drug targets for improving the outcome of corneal wound healing induced by severe injury.

TRPV4 expression has also been identified in the intact human cornea epithelium. Its activation by either a hypotonic challenge or a phorbol ester induces regulatory volume response behavior in human corneal epithelial cells [9]. Its known role as a thermosensor was documented by showing that temperatures above 25°C induced Ca^2+^ transients and outward currents [10]. Recently it was reported thatTRPV4 activation is defective in cystic fibrosis airway epithelia and contributes to induction of idiopathic lung fibrosis in mice and to transdifferentiate fibroblasts into myofibroblasts in patients [11, 12]. These recent studies prompted us to determine if TRPV4 activation by a corneal alkali burn contributes to an unfavorable wound healing response due to inflammatory fibrosis in mice.

We show here that TRPV4 activation by an alkali burn of corneas contributes to the severe fibrotic and inflammatory responses occurring during wound healing since either loss of TRPV4 gene function or intra peritoneal injection of TRPV4 antagonists reduced these responses causing improved recovery of transparency.

## Materials and Methods

Experimental protocols and the use of mice were approved by the DNA Recombination Experiment Committee and the Animal Care and Use Committee of Wakayama Medical University. Experiments were conducted in accordance with the Association for Research in Vision and Ophthalmology Statement for the Use of Animals in Ophthalmic and Vision Research.

### Alkali burn

Alkali burn of mouse corneas and conjunctiva was produced as previously reported. In brief, 3 μl of 1 N sodium hydroxide solution was applied to the right eye of 6 to 8-week-old TRPV4-null (KO) mice (RIKEN, Tokyo, Japan) [13, 14] or wild-type (WT) mice under general anesthesia [15, 16]. Ofloxacin ointment was topically administered twice a week to reduce the risk of bacterial infection. The eyes with suspicious bacterial contamination were excluded from the study. The number of eyes used in the study was 12 from WT mice and 12 from KO mice. Eye globes were processed for histology and immunohistochemistry after measurement of their diameter. In another series of experiments, 72 corneas isolated from each mouse genotype were processed for RNA extraction and real-time reverse transcription-polymerase chain reaction (RT-PCR) measured myeloperoxidase (MPO)-labeled polymorphonuclear leukocytes (PMNs) and F4/80-labeled macrophages, α-smooth muscle actin (αSMA), monocyte chemotactic protein-1(MCP-1), interleukin-6 (IL-6), vascular endothelial growth factor (VEGF) and transforming growth factor β (TGFβ), collagen Ia1 on days 5 (n = 24 WT and n = 24 KO mice), 10 (n = 24 WT and n = 24 KO mice) and 20 (n = 24 WT and n = 24 KO mice) expression after alkali burning as described below.

### Ocular fibroblast cell culture and experimentation

Outgrowth of ocular fibroblasts from eyeball shells of post-natal day 1 WT and KO mice was obtained as previously reported [7, 8]. The cells were reseeded into 60-mm dishes, grown to confluence and then treated with recombinant human TGFβ1 (1.0 ng /ml, R&D Systems, Minneapolis, MN) or vehicle control in the serum-free medium for an additional 24 h. Total RNA extracted from the cells was subjected to real time RT-PCR to determine the relative expression levels of αSMA and VEGF [7, 8]. Data were then analyzed for significance using the Mann-Whitney U test.

### Macrophage isolation and experimentation

Mouse macrophages were obtained from the peritoneal space of WT and KO mice using an oyster glycogen stimulation method as previously reported [16]. The cells were allowed to adhere to 60-mm culture dishes for 6 h in culture medium, and then non adherent cells, presumably lymphocytes, were washed out with phosphate-buffered saline (PBS). Immunocytochemistry revealed that approximately 90% of the cells obtained by this method were positive for F4/80. The culture was maintained for 6 h. The RNA extracted from the cells was analyzed by real-time RT-PCR for mRNA of MCP-1, TGFβ1, IL-6 and VEGF. Five specimens were prepared for each condition. Data were then analyzed for significance using the Mann-Whitney U test.

### Bone marrow transplantation (BMT) and ocular alkali burn

Reciprocal BMT was performed as previously reported [7, 8]. Briefly, bone marrow cells (BM cells) were obtained by flushing the tibia and femur of WT and KO mice with PBS. A total of 2 x 10^6^ WT BM cells were transplanted via tail vein infusion into recipient WT or KO mice that had received whole body irradiation of 12 Gy prior to BMT. Similarly, KO BM cells were transplanted to WT mice. The mice were subjected to alkali burn of their right eyes as described above 3 weeks after BMT. Ten days later, the animals were sacrificed and excised corneas were subjected to histological and immunohistochemical examination.

### WT mice corneal alkali burn treatment with TRPV4 antagonist

We determined if the KO phenotype of alkali burn healing is reproduced by systemic TRPV4 antagonist injection in WT mouse (n = 24). The antagonist, HC-067047 (10 mg/kg, daily) (Sigma, St. Louis, MO) [17] or its vehicle were administered *i*. *p*. daily in WT mice whose right eye corneas had been burnt with alkali. Ofloxacin ointment was topically administered twice a week to reduce the risk of bacterial infection. Eyes were then examined by using histology or immunohistochemistry on days 10 and 20 post-burn.

### Histology and immunohistochemistry

Paraffin sections (5 μm) of specimens were processed for hematoxylin and eosin (HE) staining and immunohistochemistry with antibodies (Rabbit polyclonal anti-TRPV4 antibody (1:200; Alomone, Jerusalem, Israel), Mouse monoclonal anti-α smooth muscle actin (αSMA) antibody (1:200; Neomarker, Fremont, CA), rat monoclonal F4/80 anti-macrophage antigen antibody(1:200; BMA Biomedicals, August, Switzerland), rabbit polyclonal myeloperoxidase (MPO) antibody (1:200; Neomarker, Fremont, CA) as previously reported [16].

### Real-time RT-PCR

Real-time RT-PCR was conducted by employing the TaqMan delta/delta CT method of Applied Biosystems Prism 7300 (PE Applied Biosystems, Foster City, CA) as previously reported [7, 8]. Total RNA was extracted by using a Sigma RNA extraction kit (St. Louis, MO) [7, 8]. As for examination of in vivo RNA expression levels, the corneas isolated from 4 burned mouse eyes were pooled to obtain each RNA sample. As for cell culture experiments, one RNA sample was obtained from cells in one culture dish. Real-time RT-PCR was performed. Primers and oligonucleotide probes designed by using Primers Express software (PE Applied Biosystems, Foster City, CA) (TRPV4: Mm 04499027_ml, MCP-1: Mm00441242_ml, IL-6: Mm01210732_gl, TGFβ1: Mm03024053_ml, VEGF: Mm01281447_ml, Collagen 1a1: Mm00801666_gl, MPO: Mm01298422_gl, F4/80: Mm00802524_ml, αSMA: Mm01204962_gh, GAPDH: Mm03302249_gl) were used. Data were then analyzed for significance using the Mann-Whitney U test or independent Student’s t-test.

## Results

### Documentation of TRPV4 expression

**Fig 1:**
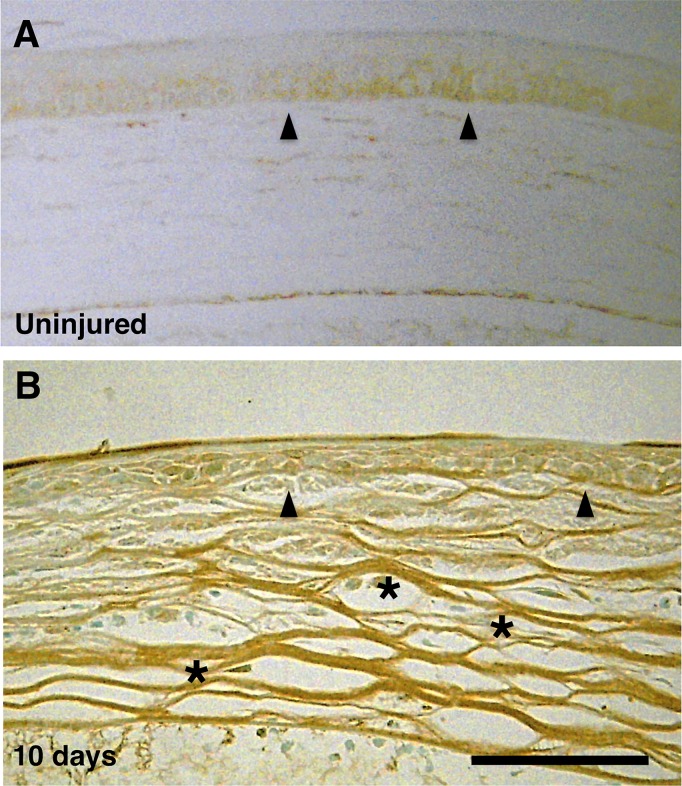
Expression of transient receptor potential vanilloid 4 (TRPV4) in corneas of wild type mice. **A:** TRPV4 expression is more preponderant in the basal epithelial cell layer (arrowheads) in the intact mouse cornea (arrowheads) with some indication of expression in the suprabasal layers. However, there is no indication of its presence in the top tear-side layer. **B:** Stromal fibroblasts undergo TRPV4 upregulation during healing at day 10 post-alkali burn in (asterisk). Scale bar = 100 μm.

TRPV4 expression was detected in the WT intact basal and suprabasal corneal epithelial layers with a validated antibody. TRPV4 was also detected in its stromal cells 10 days after being injured (Fig 1A and 1B). TRPV4 appearance in the stroma suggests that TRPV4 expression was upregulated by corneal alkali burning.

### TRPV4 deletion reduces alkali-burn induced opacification

**Fig 2:**
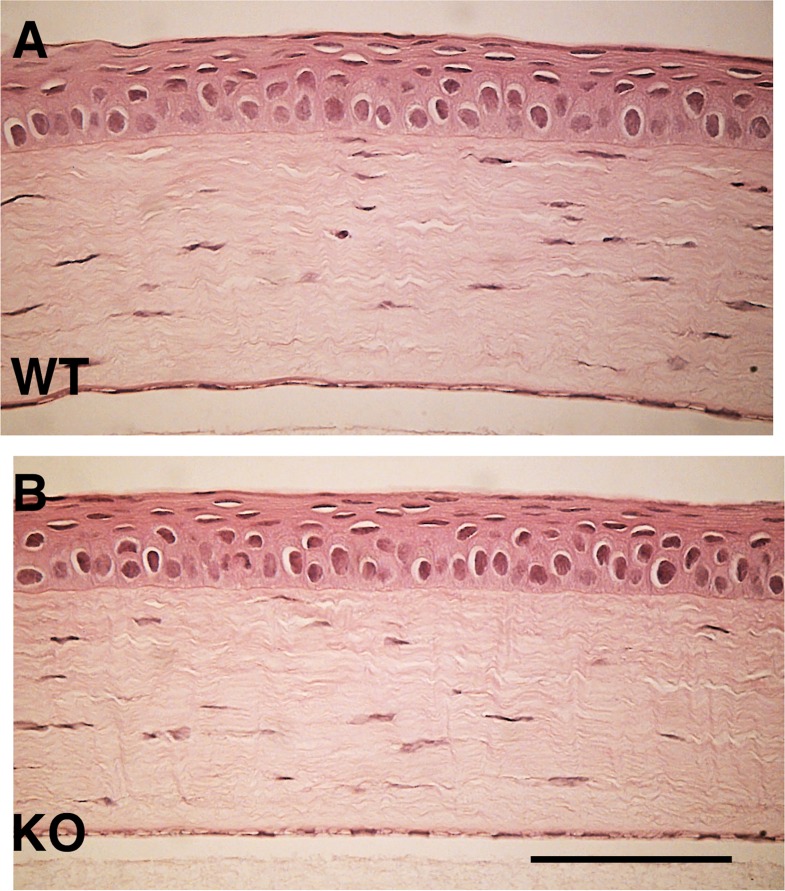
Identical histology of the wild type (WT) and TRPV4 knockout (KO) mouse corneas. There are no discernible differences in the corneal histology of WT **(A)** and **(B)** KO based on a comparison of hematoxylin-eosin (HE) staining patterns. Scale bar = 100 μm.

**Fig 3:**
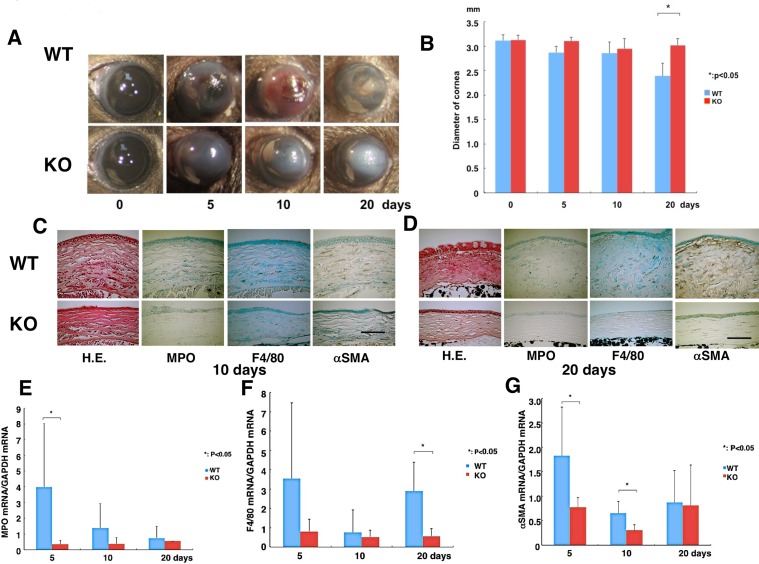
Comparison of WT and TRPV4 knockout alkali-burned corneal wound healing profiles. **A:** The corneas of wild type (WT) and TRPV4 KO mice at 0, 5, 10 or 20 days, respectively. At each time point after alkli burn, the degree of opacification in the burned cornea was more prominent in WT mice than TRPV4 KO mice. **B:** Eyeball diameter resulting from wound healing shows that WT globes are smaller at 20 days than the TRPV4 KO globes. **C and D:** Histology of burned corneas stained with hematoxylin and eosin (HE) and immunohistochemical findings at day 10 (C) and 20 (D). HE staining shows the burned cornea exhibits more severely disorganized and thicker stroma in WT corneas as compared with TRPV4 KO tissues at both day 10 (C) and 20 (D). Immunohistochemistry suggests that the density of the myeloperoxidase (MPO)-labeled polymorphonuclear neutrophils at day 10 and F4/80-labeled cells (macrophages) at 20 days is greater in a WT cornea as compared with a TRPV4 KO cornea. Healing burned corneas contain many more α-smooth muscle actin (αSMA) positive myofibroblasts in WT mice at day 10 (C) and 20 (D). Scale bar = 100 μm. **E:** Real-time RT-PCR, showed that mRNA level of MPO was less in the healing stroma of a TRPV4 KO mouse at day 5. **F:** Expression of F4/80 increased at 20 days in alkali-burned WT corneas. **G:** mRNA expression of αSMA was significantly suppressed by the loss of TRPV4 in the *in vivo* healing alkali-burned corneas on days 5 and 10, but not at day 20. Data expressed as mean ± standard error of the mean (SEM) from five specimens in each condition. *P < 0.05, Scale bar = 100 μm.

The effects of an alkali burn on wound healing outcome are compared in WT and TRPV4 gene ablated mice. Prior to wounding, light microscopic examination shown in Fig 2 did not detect any difference in their outward appearance. Alkali exposure induced very severe defective epithelial wound healing involving ulceration as well as inflammation and hemorrhage into the anterior eye chamber in the early post-burn stage followed by fibrosis/scarring-induced stromal opacification. The time dependent increases in opacification and epithelial defect development occurring in their healing phases were different from one another. In the WT corneas, epithelial defect development and opacification were more pronounced at each time point than in the KO counterpart (Fig 3A).

As corneal opacification is associated with TGFβ -induced myofibroblast transdifferentiation, we determined if there is an association between the extent of this process and changes in eyeball diameter. Such an assessment is relevant since it is indicative of myofibroblast transdifferentiation eliciting increases in contractile activity. This correlation is consistent with the more pronounced opacification and the larger contraction of the WT eyeball diameter than that in the TRPV4 KO after 20 days in the healing phase (Fig 3B).

### Injury-induced inflammatory and fibrotic mediator rises depend on TRPV4 expression

**Fig 4:**
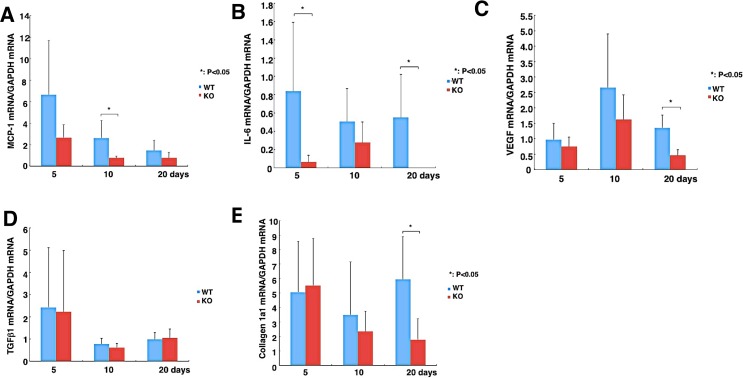
Expression pattern of wound healing-related genes in an alkali-burned cornea. **A:** Expression of monocyte-chemoattractant protein-1 (MCP-1) was transiently larger at 10 days in an alkali-burned wild-type cornea (WT) than in its TRPV4 KO counterpart but then fell to a level indistinguishable from the TRPV4 KO at 20 days. **B:** Lacking TRPV4 abolishes interleukin-6 (IL-6) mRNA expression compared to the WT at 5 and 20 days. It is noteworthy that in KO cells IL-6 gene expression was undetectable at 5 and 20 days. **C:** Difference in VEGF gene expression between WT and KO cell vascular endothelial growth factor (VEGF) mRNA reaches significance at 20 days due to loss of TRPV4 gene function. **D:** Expression level of transforming growth factor β1 **(**TGFβ1) was not affected by the loss of TRPV4 function throughout the healing interval. **E:** Expression of collagen Ia1 mRNA increases after 20 days in the healing alkali-burned WT corneas. Data represent mean ± SEM from five specimens in each condition. *P < 0.05, scale bar = 100 μm

We determined if the larger inflammatory response in WT than TRPV4 KO mice was associated with corresponding greater increases in immune cell activation and infiltration. PMN (MPO-positive) and macrophage (F4/80-positive) densities were higher in WT corneas as compared with TRPV4 KO tissues at 10 and 20 days post-alkali burn (Fig 3C and 3D). Myofibroblast generation in WT corneas increased between 10 and 20 days whereas the majority of stromal cells of TRPV4 KO alkali-burned corneas were αSMA negative. Another marked difference was that stromal swelling along with inappropriate epithelial healing were more evident in the WT than TRPV4 KO corneas (Fig 3C and 3D). Real-time RT-PCR in mRNA expression levels of both PMN and macrophage infiltration were less in the healing stroma of a TRPV4 KO mouse at day 5 or day 20, respectively, than in WT corneas (Fig 3E and 3F). Similarly, αSMA mRNA expression levels were significantly suppressed in TRPV4 KO corneas at day 5 and 10, but not at day 20 relative to the WT cornea (Fig 3G). Real-time RT-PCR analysis indicates that loss of TRPV4 function significantly suppressed MCP-1 (Fig 4A), IL-6 (Fig 4B), VEGF (Fig 4C), TGFα1 (Fig 4D) and collagen Ia1 (Fig 4E) mRNA levels at certain time point(s) throughout the wound closure interval. It is noteworthy that IL-6 upregulation was suppressed by the loss of TRPV4 function even at day 5 (Fig 4B). On the other hand, the TGFβ1 expression level was invariant despite loss of TRPV4 function throughout the healing interval (Fig 4D) whereas declines in collagen 1a1 gene expression became significant relative to WT levels after 20 days (Fig 4E). Therefore, the larger rises in immune cell infiltration in WT corneas than those in their TRPV4 KO counterpart are consistent with corresponding larger rises in the MCP-1 chemoattractant and IL-6 expression (Fig 4A and 4B). Even though myofibroblast transdifferentiation was more evident in the WT than in the TRPV4 KO corneas based on a comparison of rises in both αSMA and collagen Ia1 gene expression (Fig 4B and 4E), TGFβ1 levels remained unchanged (Fig 4 D).

### Association between changes in TGFβ -1, IL-6, VEGF and αSMA expression and TRPV4 expression

**Fig 5:**
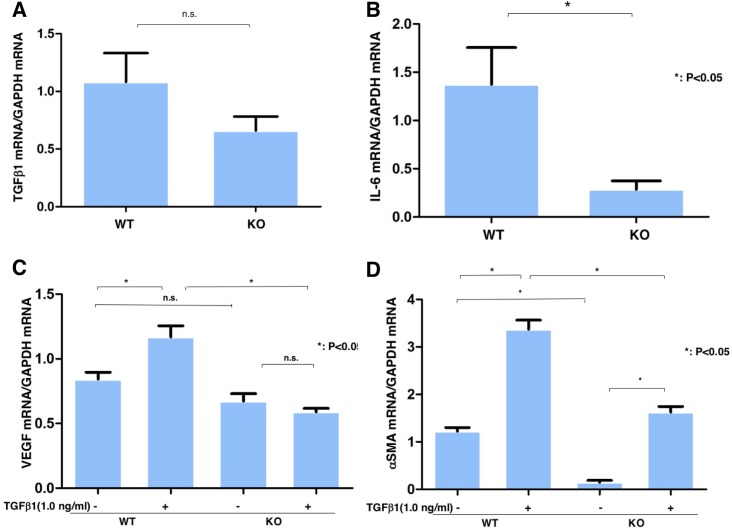
Effects of loss of TRPV4 function on TGFβ1 and IL-6 expression in ocular fibroblasts. **Comparison of effects of TGFβ1 on VEGF and αSMA expression in WT and TRPV4 KO ocular fibroblasts. A:** Loss of TRPV4 gene function does not reduce TGFβ 1 since its levels were the same irrespective of the presence or absence of TRPV4 mRNA expression. **B:** Loss of TRPV4 gene function reduces IL-6 gene expression. **C.** Exogenous TGFβ1 increases VEGF mRNA expression levels that are dependent on TRPV4 gene expression. In loss of TRPV4 function fibroblasts, TGFβ1 fails to increase VEGF gene expression. **D:** Exogenous TGFβ1 induces increases in αSMA expression irrespective of the presence or absence of TRPV4 gene expression. TGFβ1 induced rises were larger in WT ocular fibroblasts than in their TRPV4 KO counterpart. mean ± SEM *P < 0.05, Scale bar = 100 μm.

Other contributors to opacification and scarring can include inappropriate neovascularization and stromal swelling caused by formation of leaky vessels and loss of the epithelial barrier function. In order, to gain insight into how loss of TRPV4 function reduces losses in corneal transparency, we determined if TGFβ1 gene expression levels were affected by loss of TRPV4 function in orbital fibroblasts. Fig 5A shows that there was no difference between TGFβ1 expression even though myofibroblast transdifferentiation is greater in WT than TRPV4 KO fibroblasts. On the other hand, Fig 5B shows that IL-6 gene expression was markedly higher in WT than KO fibroblasts, which is consistent with its role as a chemoattractant and a proinflammatory cytokine [18, 19]. Fig 5C shows that irrespective of the presence or absence of TRPV4 expression, TGFβ1 medium supplementation did not augment VEGF gene expression. In contrast, Fig 5D shows that TGFβ1 increased αSMA expression in WT and TRPV4 KO orbital fibroblasts even though αSMA expression was much lower in TRPV4 KO fibroblasts. Both the lower αSMA expression level in TRPV4 KO cells than in its WT counterpart and the smaller increase induced by TGFβ1 show that the increase in Ca^2+^ influx required for TGFβ1 to induce increases in αSMA expression is not solely dependent on TRPV4 activation.

**Fig 6:**
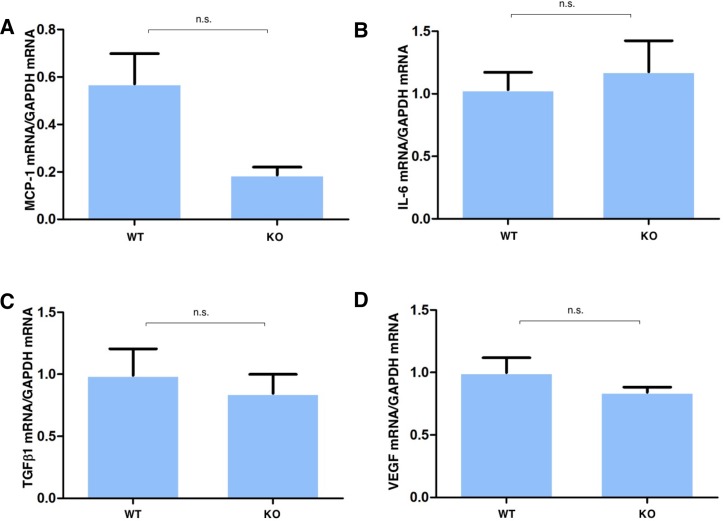
Dependence of fibrogenic gene expression on gain of TRPV4 function in peritoneal macrophages. **A:** Real-time RT-PCR showed that mRNA of MCP-1 was markedly suppressed by TRPV4 gene ablation. **B.** Difference in IL-6 gene expression between that in WT fibroblasts and TRPV4 KO macrophage was insignificant **C.** Difference in TGFβ1 gene expression between that in WT macrophage and TRPV4 KO macrophage was insignificant. **D:** Difference in VEGF gene expression between that in WT macrophage and TRPV4 KO macrophage were insignificant.

As macrophage infiltration also contributes to the inflammatory fibrotic response, we determined the effects of alkali injury on MCP-1, IL-6, TGFβ 1 and VEGF gene expression in cultured WT and TRPV4 KO macrophages. The results in Fig 6 show that MCP-1 was markedly suppressed by TRPV4 gene ablation (Fig 6A), but those for IL-6 (Fig 6B), TGFβ1 (Fig 6C) and VEGF (Fig 6D) were not affected in cultured macrophages.

### BMT identifies origins of cells affecting corneal injury response

**Fig 7:**
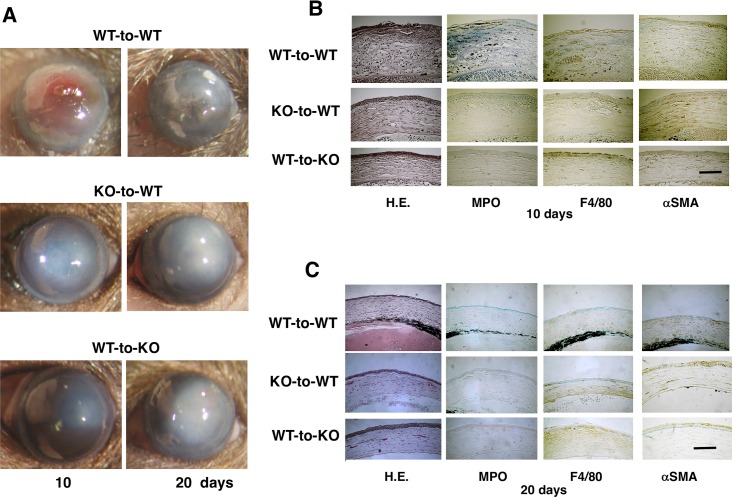
Time dependent corneal alkali burn-induced wound healing responses in mouse chimeras receiving bone marrow transplantation (BMT). **A:** The corneal response to an alkali exposure in chimera mice (WT-to-WT, KO-to-WT and WT-to-KO group) 10 and 20 days after alkali burn. Inflammation (distribution of MPO-labeled PMNs and F4/80-positive macrophages) and myofibroblast distribution (a tissue fibrosis marker) in corneal stroma of chimeric mice of the WT-to-KO and KO-to-WT groups seemed less severe as compared with those in the WT-to-WT group mice. **B and C:** HE stained histology shows that increases in cell density in the swollen stroma of a WT-to-WT cornea as compared with a KO-to-WT and a KO-to-WT tissue after 10 and 20 days. Immunohistochemistry shows that the cornea of a WT-to-KO and KO-to-WT mouse has less stromal αSMA staining as well as lower levels of MPO and F4/80 immunoreactivity as compared with the mice undergoing WT-to-WT transplantation 10 and 20 days post-alkali burn. Scale bar is 100 μm.

TRPV4 gene function loss is associated with a decline in alkali-induced stromal fibroblast transdifferentiation into myofibroblasts (Fig 3G) as well as suppression of MCP-1 and VEGF expression (Fig 4A and 4C). Accordingly, we examined if the improved wound healing outcome in KO mice is attributable to loss of TRPV4 gene expression on resident corneal cells and/or infiltrating inflammatory cells. To answer this question, we employed *in vivo* chimera mice generated by reciprocal bone marrow transplantation (BMT). Using WT and TRPV4 KO mice, two different paradigms were used to determine the origins of infiltrating inflammatory cells in eliciting the TRPV4 KO healing phenotype in response to injection of KO corneal alkali burn bone marrow isolates into WT recipients (KO-to-WT), conversely WT bone marrow isolates were injected into- KO (WT-to-KO) and WT mice received WT bone marrow isolate (WT-to-WT). Duration of healing delay, severity of inflammation and corneal opacification in WT-to-KO and KO-to-WT were less than that in WT-to-WT group (Fig 7A). Immunohistochemistry showed that the cornea of a WT-to-KO and a KO-to-WT chimera mouse had less stromal αSMA staining as well as lower levels of macrophage marked F4/80 and polymorpholeucocyte (PMN) marked MPO immunoreactivity as compared to a WT-to-WT mouse at 10 and 20 days (Fig 7B and 7C). These findings are consistent with the notion that TRPV4 gene expression activation in both BM -derived cells and in tissue resident cells contribute to alkali burn-induced inflammatory responses and myofibroblast transdifferentiation during the compromised corneal wound healing process.

### Systemic TRPV4 antagonist injection reduces alkali-induced corneal inflammatory fibrosis

**Fig 8:**
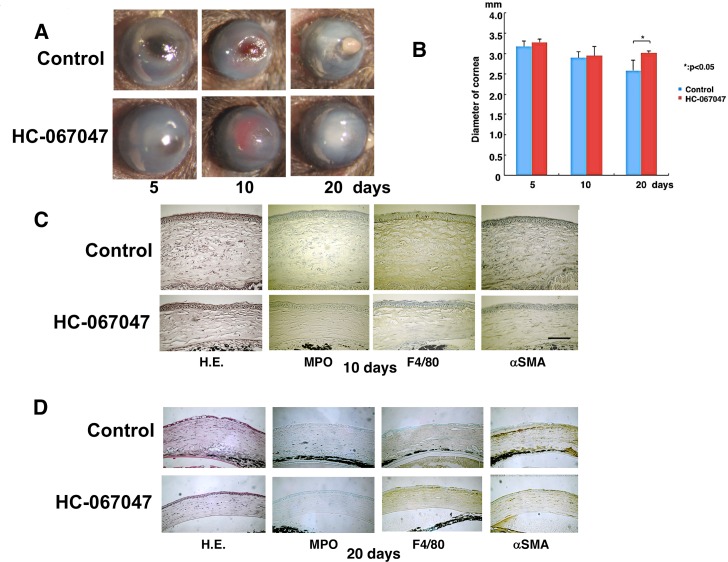
TRPV4 antagonist (HC-067047) injection attenuates corneal inflammatory fibrosis induced by alkali burn in wild-type (WT) mice. **A.** The corneas of WT mice treated with or without HC-030031 at 5, 10 or 20 days. Corneal transparency restoration is markedly improved in the mice treated with a TRPV4 antagonist HC-067047 at each time point. **B.** Eyeball diameter during wound healing after alkali burn shows untreated globes are smaller at 20 days than antagonist treated globes. **C and D.** Histology of burned corneas stained with HE and immunohistochemical stained at day 10 (C) and 20 (D). The stromal organization is more disorganized in untreated mice cornea than in antagonist treated mice. The antagonist treated mice cornea has lower levels of infiltration of MPO-labeled neutrophils and F4/80-positive macrophages as well as less marked αSMA staining at each time point. Scale bar is 100 μm.

To assess if TRPV4 antagonist treatment of WT alkali burned corneas reduces inflammation and fibrosis, mice received daily intraperitoneal injections of a TRPV4 antagonist, HC-067047 (10 mg/kg). Corneal transparency restoration was markedly improved and it had an appearance similar to that seen in alkali burnt TRPV4 KO mice. Consistent with less myofibroblast transdifferentiation in TRPV4 KO mice, antagonist treatment after 20 days reduced globe diameter shrinkage more markedly than in the untreated WT mice (Fig 8A and 8B). Another indication of TRPV4 involvement in mediating inflammation is that the antagonist-treated mice exhibited lower levels of MPO and F4/80 infiltration as well as less marked αSMA myofibroblast staining as compared to mice treated instead with the control vehicle at 10 and 20 days (Fig 8C and 8D). These findings indicate that TRPV4 antagonist treatment improves the wound healing outcome resulting from a corneal alkali burn in mice (Fig 8C and 8D).

## Discussion

We show here that TRPV4 activation contributes to the severe inflammatory fibrosis resulting from injuring mouse corneas with alkali. Its involvement in this process is evident since in TRPV4 KO mice this maladaptive response is less severe than that in their WT counterpart. Additional evidence for TRPV4 involvement was obtained by showing that the severity of the inflammatory fibrosis was attenuated to a similar extent following systemic daily administration of a TRPV4 antagonist, HC-067047, for 20 days. Interestingly, we also found that the severe inflammatory fibrotic response that occurs in the WT is attributable to TRPV4 activation on both corneal resident cells and infiltrating activated immune cells. Such activation induced inflammatory fibrosis through time dependent changes in immune cell infiltration and the expression of chemoattractant as well as proinflammatory cytokines that are unique to increases in TRPV4 activity in the cornea. Taken together, these results suggest that additional studies are warranted to further evaluate in other species if TRPV4 antagonist treatment of corneal alkali burns can improve restoration of corneal function during wound healing by reducing inflammatory fibrosis.

Based on the results of immunohistochemistry and real-time RT-PCR, alkali-induced inflammation development involved PMN infiltration at day 5 followed by macrophage influx at 20 days in WT mice. Such effects were suppressed by TRPV4 gene deletion. A similar phenotype of decreased PMN infiltration and less severe edema were reported in an experimental lung injury in a TRPV4 KO [20–24]. We found that the rises in IL-6 expression that occurred in the WT mice were markedly suppressed by the loss of TRPV4 as early as day 5. This decline in vivo may be attributable to depressed mesenchymal cell IL-6 release since IL-6 accumulation was also markedly less in KO than WT ocular fibroblasts (Fig 5B). This difference suggests that TRPV4 induced IL-6 release contributes to the more pronounced inflammatory response in WT mice since it is well established that IL-6 besides being a chemoattractant plays a critical role in amplification of tissue inflammation. Accordingly, the decline in IL-6 in the absence of TRPV4 expression is consistent with less inflammation in these mice than in their WT counterpart [25]. Since macrophages express significant TRPV4 levels, the delay in macrophage infiltration seen at day 20 due to declines in MCP-1 is attributable to loss of TRPV4 gene function (Fig 4A) [26]. Such delay, in combination with reduced IL-6 levels in the TRPV4 KO tissue, appears to underlie less ocular inflammation. Furthermore, TRPV4 gene ablation also suppressed MCP-1 expression levels, which is a critical macrophage chemoattractant. Such suppression is consistent with the delay in macrophage infiltration into the injured stroma, but may not fully account for this change since macrophage infiltration into the damaged stroma might also be influenced by a growth factor/cytokine complex elaborated by PMNs and resident keratocytes/myofibroblasts.

The loss of TRPV4 gene function also reduced VEGF expression levels in addition to declines in both IL-6 and MCP-1. As noted, at day 5 alkali injury-induced upregulation of IL-6 expression was already markedly suppressed in the injured tissue, which was followed by declines in the MCP-1 and VEGF mRNA expression levels. As injury is known to induce to rises in IL-6 release, a decline in PMN infiltration is consistent with reduced IL-6 release in the early injury phase in TRPV4 KO tissue [25, 27]. The reductions in MCP-1 and VEGF mRNA levels in the injured KO tissue are consistent with lower gene expression of these factors in TRPV4 KO ocular fibroblasts than in their WT counterpart. The decline in VEGF mRNA levels in KO tissue at day 20 is partially consistent with VEGF mRNA expression lowering in cultured ocular KO fibroblasts in the presence or absence of exogenous TGFβ1. Its level was more substantially depressed by TRPV4 gene knockout in cultured macrophages, which may be sufficient to account for the fall in macrophage infiltration in KO tissue at day 20. Even though we reported earlier that rises in TGFβ1 mRNA expression induced by TRPV1 and TRPA1 activation with alkali elicit fibrogenesis in the healing mouse cornea, we show here that TGFβ1 mRNA expression was unaffected by loss of TRPV4 gene function. Such invariance *in vivo* agrees with no significant difference between TGFβ1 mRNA expression in cultured TRPV4 KO and WT ocular fibroblasts as well as corresponding macrophages.

αSMA expression is a well accepted hallmark of tissue fibrosis underlying transdifferentiation of fibroblasts into myofibroblasts [7, 8, 15, 28, 29]. This phenomenon is modulated positively or negatively by various growth factors. Myofibrolast transdifferentiation leads to excess expression of extracellular matrix components and stromal remodeling. Indicative of these changes are rises in collagen type I and scarring during wound healing [28]. In the current in vitro study, the loss of TRPV4 gene function attenuated myofibroblast generation and collagen 1a1 expression. Although the in vivo TGFβ1 level was not affected by the loss of TRPV4 in healing corneas, the decline in myofibroblast transdifferentiation may be due to impaired cognate receptor function or signal transduction coupling inducing transactivation of other Ca^2+^ influx pathways besides TRPV4. This is possible since there are numerous other Ca^2+^ influx pathways besides TRPV4 which can be activated to induce increases in Ca^2+^ influx [30]. The possible involvement of different Ca^2+^ influx pathways besides TRPV4 may be a factor contributing to the variable effects of TGFβ receptor activation inducing responses accompanying transdifferentiation of fibroblasts into myofibroblasts. If their involvement is not consistent, TGFβ receptor-induced TRPV4 transactivation may not in all cases maximally activate the linked signaling pathways mediating this response. Another contributing factor may be that variable declines in IL-6 expression in TRPV4 KO tissue could contribute to inconsistent declines in myofibroblast transdifferentiation. Such a possibility agrees with a report showing that myofibroblast differentiation is not accelerated by exogenous IL-6, but is supported by endogenous IL-6 expressed by the cells themselves [31].

Impairment of myofibrobalst differentiation in TRPV4-null cultured ocular fibroblasts could be explained by the current finding that expression of IL-6 was suppressed in TRPV4 KO cells. On the other hand, in a mouse model of idiopathic lung disease, TRPV4-linked signaling contributes to tissue fibrosis via TGFβ-dependent signaling [12]. This result differs from the current study which found that TGFβ1 expression was unaffected by TRPV4 gene ablation suggesting tissue specific differences in TRPV4-dependent modulation of local inflammation and fibrosis.

Using mouse chimeras demonstrated that the improved KO healing phenotype is attributable to the absence of TRPV4 expression in both corneal resident cells (mesenchymal cells) and infiltrating macrophages. Reciprocal BMT transplants, involving WT or KO mice, receiving KO or WT bone marrow, respectively indicate that alkali exposure induced less inflammation in the KO than their WT counterpart (c.f. Fig 7). This difference in wound healing outcome is evident based on gross corneal appearance as well as histology and declines in PMN and macrophage immunohistochemical staining along with αSMA immunostaining on days 10 and 20 following injury.

Taken together, TRPV4 gene ablation reduces both alkali-burn -induced inflammatory reaction and subsequent fibrosis in mouse corneas. Similar findings of TRPV4 receptor signal involvement in inducing tissue inflammation were reported in the gastrointestinal tract and joints [32]. Lung fibrosis was also attenuated by the loss of TRPV4 gene function [12]. The present results suggest that chemical blocking of TRPV4 channel activation could be beneficial in treating inflammation-based corneal diseases. To test this possibility, we determined that systemic *i*. *p*. administration of a TRPV4 antagonist improved the wound healing outcome in alkali-burned mouse corneas. Such improvement is comparable to declines in fibrosis and inflammation in TRPV4 KO mice. This correspondence suggests TRPV4-drug targeting may provide a novel strategy to treat chronic inflammation and resultant fibrosis/opacification caused by severe corneal injury.

## Supporting Information

S1 Fig Fibrosis inflammatory biomarker absence in uninjured TRPV4 KO corneas Immunohistochemical expression profiles of PMN, macrophage and myofibroblast biomarker indicate that neither fibrosis nor immune cell activation occurs in uninjured WT and TRPV4 KO corneas. Fibrosis and inflammatory induction by chemical injury shown in Fig 3 are dependent on TRPV4 expression since loss of its expression markedly reduced the time dependent increases in myeloperoxidase (MPO)-labeled polymorphonuclear neutrophils, F4/80-labeled cells (macrophages) and α-smooth muscle actin (αSMA) expression to levels that were nearly indistinguishable from those shown in this figure at T = 0 point in uninjured WT and TRPV4 corneas. Scale bar is 100 μm.
(TIF)

S1 File This is the excel file of Fig 3E. (XLS)

S2 File This is the excel file of Fig 3F. (XLS)

S3 File This is the excel file of Fig 3G. (XLS)

S4 File This is the excel file of Fig 4A. (XLS)

S5 File This is the excel file of Fig 4B. (XLS)

S6 File This is the excel file of Fig 4C. (XLS)

S7 File This is the excel file of Fig 4D. (XLS)

S8 File This is the excel file of Fig 4E. (XLS)

S9 File This is the excel file of Fig 5A. (XLS)

S10 File This is the excel file of Fig 5B. (XLSX)

S11 File This is the excel file of Fig 5C. (XLS)

S12 File This is the excel file of Fig 5D. (XLS)

S13 File This is the excel file of Fig 6A. (XLS)

S14 File This is the excel file of Fig 6B. (XLS)

S15 File This is the excel file of Fig 6C. (XLS)

S16 File This is the excel file of Fig 6D. (XLS)

S17 File This is the excel file of Fig 3B. (XLS)

S18 File This is the excel file of Fig 8B. (XLS)
