# Supplementary figures and images for: Loss of TRPV4 Function Suppresses Inflammatory Fibrosis Induced by Alkali-Burning Mouse Corneas
Source: PLoS One. 2016 Dec 28;11(12):e0167200. doi: 10.1371/journal.pone.0167200 (PMC5193391; doi:10.1371/journal.pone.0167200)

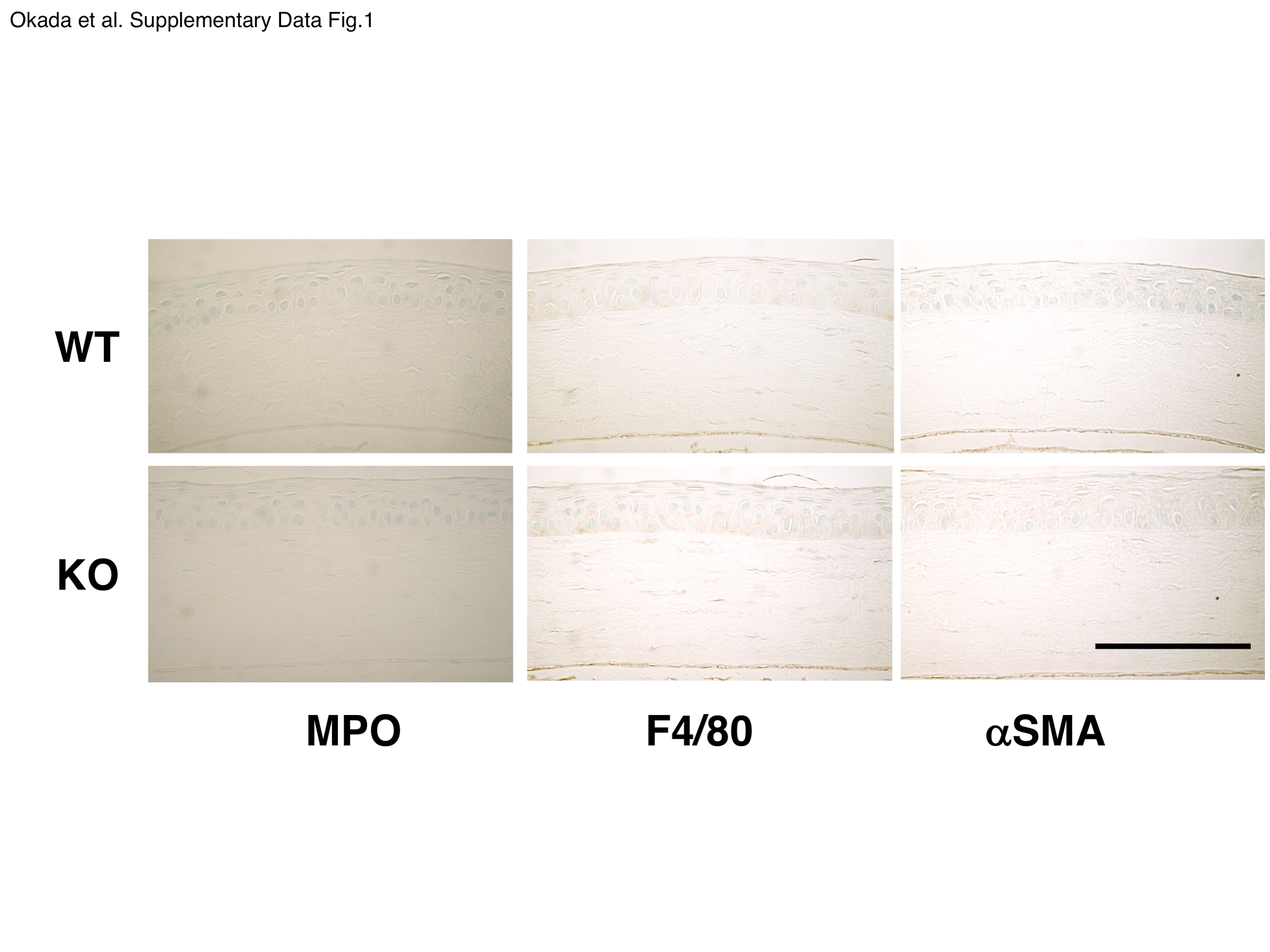

Supplement: S1 Fig — Fibrosis and inflammatory induction by chemical injury shown in Fig 3 are dependent on TRPV4 expression since loss of its expression markedly reduced the time dependent increases in myeloperoxidase (MPO)-labeled polymorphonuclear neutrophils, F4/80-labeled cells (macrophages) and α-smooth muscle actin (αSMA) expression to levels that were nearly indistinguishable from those shown in this figure at T = 0 point in uninjured WT and TRPV4 corneas. Scale bar is 100 μm. (TIF) [file pone.0167200.s001.tif]
